# The Use of Environmental Enrichments Affects Performance and Behavior of Growing Rabbits Housed in Collective Pens

**DOI:** 10.3390/ani9080537

**Published:** 2019-08-07

**Authors:** Angela Trocino, Cristina Zomeño, Eirini Filiou, Marco Birolo, Peter White, Gerolamo Xiccato

**Affiliations:** 1 Department of Comparative Biomedicine and Food Science (BCA), University of Padova, Viale dell’Università 16, Legnaro, I-35020 Padova, Italy; 2 Department of Agronomy, Food, Natural Resources, Animal and Environment (DAFNAE), University of Padova, Viale dell’Università 16, Legnaro, I-35020 Padova, Italy; 3 Sydney School of Veterinary Science, Faculty of Science, B19, R.M.C. Gunn Building, The University of Sydney, Sydney, NSW 2006, Australia; 4 Sydney Institute if Agriculture, B19 R.M.C. Gunn Building, The University of Sydney, Sydney, NSW 2006, Australia

**Keywords:** group housing, platform, tube, growth, behavior, reactivity

## Abstract

**Simple Summary:**

Group housing of growing rabbits is currently used in commercial farms to improve animal welfare. However, these systems have shown some weaknesses, mainly associated with the aggressive behavior exhibited among rabbits. Environmental enrichment can offer additional space and a sheltered area in which an animal can protect itself from pen mates, but studies in rabbits under farming conditions are scarce. Thus, this study evaluated the use of two types of enrichment (an elevated plastic-slatted platform and/or a plastic hiding tube) in rabbits housed in collective pens within large groups during the growing period. The use of the platform allowed rabbits to move up/down, to rest in a more comfortable position and to increase explorative behavior, without modifying production performance. However, more injured rabbits were found at the end of the trial in pens with platforms. The presence of the tube impaired growth performance and did not modify behavior. Hence, elevated platforms have been shown to work as a structural enrichment in group-housed rabbits, whereas the usefulness of the tube remains questionable.

**Abstract:**

This study assessed the effects of an elevated plastic-slatted platform and/or a plastic hiding tube in collective pens with large group sizes (27 or 36 rabbits/pen; 16 rabbits/m^2^) on the performance and welfare of rabbits kept from weaning (at 33 days of age) to slaughter (at 68 or 75 days of age). Growth performance, injuries, and behavior (video recorded for 24 h) of rabbits (*n* = 504) were recorded. The platform allowed rabbits to adopt the rearing position more frequently (+0.14 events during 2 min every 30 min across 24 h) and to rest with stretched body for longer (+3.8% of observed time) (*p* ≤ 0.001). Production parameters and reactivity at the open field test were not modified, but the occurrence of injured rabbits at the trial end was higher in pens with platforms (+8.9%; *p* ≤ 0.01). This result was possibly related to the higher group size in pens with platforms (36 rabbits) compared to those without platforms (27 rabbits). The inclusion of the tube decreased growth (−2.2 g/d; *p* ≤ 0.05), whereas it was scarcely used by rabbits and it did not substantially change their behavior or the occurrence of injuries. In conclusion, under the experimental conditions of this study, elevated platforms worked as a useful structural enrichment in view of animal behavior but negatively impacted on the rate of injuries, whereas the usefulness of the tube was not confirmed.

## 1. Introduction

To improve animal health and welfare in rabbit farms, the European Parliament has requested a change from conventional cages to collective pens, also called parks [1,2]. Some member countries, even if not producing countries, have their own legislation or recommendations on the housing of farmed rabbits (i.e., Austria, Belgium, Germany, Hungary, Italy, The Netherlands). Some of them are sustaining a transition towards rearing systems with large groups of animals kept in open top elevated pens with platforms and slatted floors. Nevertheless, these housing systems have been developed over the last decade, but are not yet in widespread use [3] nor fully validated under commercial conditions due to technical and economic constraints. Standards are lacking and their adoption would imply the renewal of rabbit production all over Europe, i.e., changes of farm facilities and management and high investment costs in a small and economically weak sector.

Undoubtedly, in comparison with bicellular (2 rabbits) or multi-purpose (4–6 rabbits) cages, collective pens can provide rabbits with more opportunities to satisfy their ethological needs, with special emphasis on free social contacts and increased total available space for movement [4,5]. In rabbits, social relationships start from the beginning of their life, but in the wild, before sexual maturity, almost 100% of young males and 50% of young females leave the original group [6]. Under farming conditions, when sexual maturity is approaching, i.e., after 9–10 weeks of age depending on breed and genotype, aggression occurs among growing rabbits kept in groups, which could result in skin lesions and wounds and, consequently, in welfare impairment [6,7].

A group size higher than 7–9 growing rabbits (the usual litter size at weaning) increases the risk of aggression and wound rates as well as the risk of disease spread [6], findings that are proven by the latest research [2,8,9]. Conversely, when the stocking density recommended by the European Food Safety Authority (EFSA, 2005) is applied (16 rabbits/m^2^ at 2.5 kg slaughter weight; 40 kg slaughter weight/m^2^) [6], the small group size limits the space available (about 0.5 m^2^) for rabbit movements in each pen/park. That is why, under commercial conditions, collective systems are moving towards the use of large groups in large pens and research has focused on how to manage aggression in these systems.

Therefore, environmental and structural enrichments of housing systems could provide animals with the opportunity to display different behaviors, reduce abnormal behaviors, limit aggression and minimize animal stress. Among the different enrichments, gnawing materials are most commonly used. In growing rabbits kept in cages and pens with both small (2–4 animals/cage) and medium-size (13–15 animals/pen) groups, gnawing sticks have often reduced stereotyped behaviors, such as cage bar biting or chewing [10,11], aggressive interactions [12], skin wounds [13], and ear lesions [14]. On the other hand, some studies have not found a significant reduction of abnormal behaviors [15], whereas others have reported an increase of aggressive behavior when gnawing materials were provided [13].

The use of structural enrichments, such as elevated platforms or hiding elements (e.g., tubes or boxes) to offer a shelter and/or more possibilities to escape in case of aggression, has received less attention in rabbits kept in large groups. The presence of elevated platforms can offer additional space for movement and for physical exercise, without impairing productivity and health [16,17,18], even if the platform surface can or cannot be included in the total available surface depending on national regulations. On the other hand, the use of different hiding elements in reproducing does kept in groups [19] or in adult laboratory rabbits [20] has not resulted in a significant reduction of aggression among animals or in the provision of an effective site for shelter and resting.

In this context, the present study aimed at evaluating the effect of two structural elements (an elevated plastic-slatted platform and/or a plastic hiding tube) on the growth performance, occurrence of lesions, behavior, and reactivity at the open field test in growing rabbits kept in large collective pens (27–36 rabbits/pen) at the same stocking density (16 rabbits/m^2^) and slaughtered at 10 or 11 weeks of age.

## 2. Materials and Methods 

The study was approved by the Ethical Committee for Animal Experimentation of the University of Padova (Italy) (Project number 14/2012, approved on 15 February 2012). All animals were handled according to the principles stated in the EC Directive 2010/63/EU regarding the protection of animals used for experimental and other scientific purposes. 

### 2.1. Animals, Housing and Experimental Design

The trial was performed at the experimental farm of the University of Padova (Italy). Temperatures ranged between 13 °C and 24 °C and a natural photoperiod (11–13 h daylight) was used.

**Figure 1:**
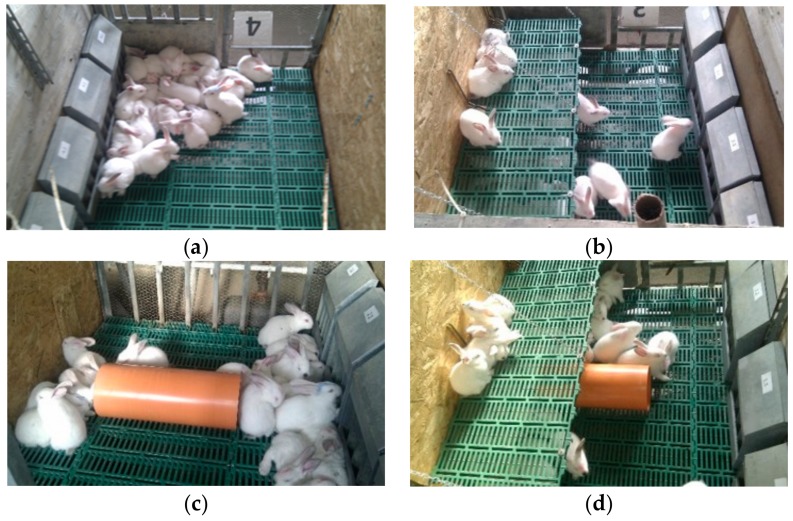
Photographs of pens used to evaluate enrichment with platforms and tubes: (**a**) pens without enrichment; (**b**) pens with platform; (**c**) pens with tube; (**d**) pen with platform and tube.

At weaning (33 days of age), a total of 504 Hyplus rabbits (Hypharm, Groupe Grimaud, Roussay, France) of both sexes were selected from healthy and homogenous litters in a commercial farm and moved to the experimental farm of the University. Rabbits were individually identified by ear mark and housed in 16 open-top pens (120 cm length × 140 cm width; 1.68 m^2^) equipped with a plastic-slatted floor (rectangular holes: 7 cm length × 1 cm width; distance between the holes: 0.7 cm) at a stocking density of 16 animals/m^2^. The sidewalls of the pens (105 cm height) were made of wooden material with the front/back walls of galvanized wire net. Each pen was equipped with four manual feeders and four automatic nipple drinkers (Figure 1). 

Two types of enrichment, a plastic-slatted elevated platform (120 cm length × 50 cm width and 30 cm above the floor; 0.60 m^2^) and a plastic tube (20 cm diameter; 50 cm length), were used to obtain four types of collective pens (Figure 1):

1. pens without enrichment (4 pens; 27 rabbits/pen; 16 rabbits/m^2^) (Figure 1a);
2. pens with platform (4 pens; 36 rabbits/pen; 16 rabbits/m^2^) (Figure 1b);
3. pens with tube (4 pens; 27 rabbits/pen; 16 rabbits/m^2^) (Figure 1c);
4. pens with platform and tube (4 pens; 36 rabbits/pen; 16 rabbits/m^2^) (Figure 1d).

The number of rabbits in pens with platforms was increased to maintain the same stocking density. Within each pen type, half of the animals (2 pens) were slaughtered at 68 days of age and the remainder (2 pens) at 75 days of age.

The animals were provided with ad libitum access to fresh water and to a pelleted commercial diet (dry matter: 89.4%, crude protein: 15.1%; ether extract: 3.9%; neutral detergent fiber: 38.1%; acid detergent fiber: 22.0%; and acid detergent lignin: 6.5%) without coccidiostat. The diet was formulated to meet the nutritional requirements of growing rabbits according to de Blas and Mateos [21].

### 2.2. Growth Performance Evaluation

Individual live weight and pen feed intake were recorded twice per week. Health status was monitored daily to detect any clinical signs of diseases. The day before slaughter, all rabbits were individually examined for the presence of injuries (i.e., multiple scratches, open or scabbed wounds) due to aggression. 

### 2.3. Behavioral Evaluation

The behavior of the rabbits was video-recorded for 24 h at 63 days (all pens) and at 70 days (8 pens housing rabbits to be slaughtered at 75 days). Two minutes per half-hour were recorded for evaluating the duration of some behaviors and all occurrences of other behaviors as reported in Table 1 [22,23,24]. During the night, minimal light (15 lux) was used to avoid disturbing the rabbit’s activities. Nutrition behaviors (feeding and drinking) were expressed as a percentage of the total observation time. Resting, social, explorative and locomotor behaviors were expressed as a percentage of time spent on these behaviors when rabbits were visible, i.e., excluding the time spent feeding, drinking, under the platform and/or inside the tube. The occurrence of rearing, hops and aggressive interactions was expressed as the number of events per pen per observation interval (n).

### 2.4. Open-Field Test

The open-field test was performed to evaluate rabbits reactivity towards a new environment at 65 days of age on 80 rabbits (10 animals from each of 8 pens for rabbits to be slaughtered at 68 d) and at 72 days of age on another 80 rabbits (10 animals from each of 8 pens for rabbits to be slaughtered at 75 days). Within pens, tested animals were homogeneously distributed by sex. 

The test was conducted in an arena (2 m × 2 m) with 0.8-m-high wooden walls and plastic floor divided into 9 numbered squares. The arena was in a contiguous room in the same stable where the trial was conducted. The total duration of each test was 10 min per animal. Each rabbit was put in a closed wooden box (22 cm length × 30 cm width × 30 cm high) connected to the arena by a sliding door. After one minute, the sliding door was opened and the number of attempts (n) the rabbit made and the time (latency, seconds) spent to enter the arena was recorded for another minute. If, after this minute, the rabbit was still in the box, it was gently pushed into the arena, the sliding door was closed and the behavior of the rabbit was video-recorded for 8 min. The behaviors that were evaluated during the open-field test are described in Table 2 [24,25,26]. 

### 2.5. Statistical Analysis

All data were analyzed with SAS 9.4 software (SAS Institute Inc., Cary, NC, USA). The performance traits (live weight at 33 days, live weight at slaughter, weight gain per day, feed intake per day and feed conversion) were analyzed separately using the GLM procedure and fitting a generalized linear model with platform (no vs. yes), tube (no vs. yes), slaughter age (68 days vs. 75 days) and their interactions as the main factors, and the pen as the experimental unit. Injury data were coded as a binary variable (0 = no injuries, 1 = injuries of any score) for estimating the percentage of animals with injuries using the CATMOD procedure by maximum likelihood analysis with platform, tube, slaughter age and their interactions as main factors.

The different behaviors detailed in Table 1 and evaluated across 24 h were analyzed separately with a generalized linear mixed model using the GLIMMIX procedure with platform, tube, animal age (63 days vs. 70 days), and their interactions as fixed effects and with the observation hour as a random effect. Data from the same pen were considered as repeated measures. An underlying Poisson distribution was assumed for all data.

Data from the open-field test was analyzed using the GLIMMIX procedure with a generalized linear mixed model considering platform, tube, animal age (65 days vs. 72 days), and their interactions as fixed effects, and pen as the random effect. An underlying Poisson distribution was also assumed for these data. The percentage of animals entering the arena spontaneously was coded as a binary variable (Enter = Yes or No) and evaluated by maximum likelihood analysis with the CATMOD procedure which used platform, tube, animal age, and their interactions as the main factors.

Overall, differences among means with a *p*-value ≤ 0.05 were accepted as representing statistically significant differences. The complete sets of *p*-values for the single effects and their interactions are provided in Supplementary Tables S1–S3. Supplementary Table S4 provides descriptive statistics of rabbit behavior registered across 24 h according to the presence (no or yes) of enrichments (platform and tube) and animal age at recordings. Supplementary Table S5 provides descriptive statistics of reactivity of rabbits during the open-field test according to the presence of enrichments and animal age at recordings.

## 3. Results

### 3.1. Effect of Enrichment Type

The presence of the platform did not affect growth performance, however at the end of the trial a higher rate of rabbits with injuries was scored in pens with a platform compared to those without (+8.9 percentage points; *p* ≤ 0.01) (Table 3). 

The presence of a hiding tube reduced daily weight gain (−2.2 g/day; *p* ≤ 0.05) (Table 3). Even when differences were not statistically significant, a higher rate of injured rabbits (+4.2 percentage points) was also observed in pens with a tube.

With regard to the use of the platforms, rabbits were found on the platform for a mean of 20.6% of the observation time (minimum = 5.55%; maximum = 49.6%; standard deviation = 6.78%) (intervals of 2 min every 30 min across 24 h) and under the platform for an mean of 29.4% of the observed time (minimum = 8.52%; maximum = 50.0%; standard deviation = 8.26%). In addition, they moved 0.95 times per observation interval to/from the platform (minimum = 0.00; maximum = 8.00; standard deviation = 1.33) (data not shown in tables). Regarding other behaviors (referring to the observation time the animals were visible and excluding time spent in nutrition behavior), rabbits were observed for a longer time resting in a stretched position (+3.8 percentage points; *p* ≤ 0.001) and biting or licking the pen elements (+0.20 percentage points; *p* ≤ 0.01) (Table 4) in pens with platforms compared to those without. They also adopted the rearing position more frequently (+0.14 events per observation interval; *p* ≤ 0.001). The differences in resting time with stretched body were higher at 70 d (28.7% vs. 33.5% of observation time in pens without vs. those with a platform) (platform × age interaction, *p* ≤ 0.001). The time the rabbits were observed drinking (−0.46 percentage points; *p* ≤ 0.01), allo-grooming (−0.49 percentage points; *p* < 0.001), and moving around the pen (−0.20 percentage points; *p* ≤ 0.05), as well as the number of aggressive encounters (−0.11 events per interval; *p* ≤ 0.01) were all lower in pens with platforms compared to those without (Table 4). Time spent self-grooming was also lower in pens with a platform compared to those without, but only at day 63 (platform × age interaction, *p* ≤ 0.001) (Table 4). Rabbits were found inside the tube for a mean of 2.4% of the total observation time (minimum = 0.00%; maximum = 12.9%; standard deviation = 3.18%) (data not shown in tables). The use of the tube did not modify the time spent for nutrition or other behaviors (Table 4). Rabbits kept in pens with a tube compared to those without showed a lower number of rearing positions (−0.11 events per observation interval; *p* ≤ 0.001) (Table 4). 

Some differences were recorded for the open-field test including the time spent grooming which was higher (+2.18 s) in rabbits from pens with a platform compared to those without (*p* ≤ 0.05) (Table 5). The use of the tube did not modify reactivity during the open-field test (Table 5).

Regarding the interaction between the two types of enrichment, rabbits were observed inside the tube for 4.6% of total observation time in pens fitted with only a tube (minimum = 0.00%; maximum = 12.9%; standard deviation = 3.18%) compared to 0.25% of the time in pens with both a tube and platform (minimum = 0.00%; maximum = 5.28%; standard deviation = 0.70%) (data not shown in tables). Moreover, those rabbits housed in pens with any form of enrichment (platform, tube, or both items) were observed sniffing and moving for a lesser time period compared to those from pens with no enrichment (interaction platform × tube, 0.001 ≤ *p* ≤ 0.05) (Table 4).

### 3.2. Effect of Animal Age

When slaughter age increased from 68 to 75 days, animals exhibited a higher final live weight (+219 g; *p* ≤ 0.001), a lower daily weight gain (−2.4 g/day; *p* ≤ 0.05) together with an increased feed intake (+6 g/day; *p* ≤ 0.001), which impaired the feed conversion index (+0.36; *p* ≤ 0.001) (Table 3).

In terms of behavior, when age was increased, rabbits were observed for a longer time period resting in the crouched position (+6.0 percentage points), self-grooming (+5.3 percentage points), and sniffing (+1.81 percentage points) (*p* ≤ 0.001), at the expenses of resting in the stretched position (−12.8 percentage points) (*p* ≤ 0.001) and allo-grooming (−0.15 percentage points; *p* ≤ 0.05) (Table 4). Moreover, the number of rearing movements (−0.14 events per observation interval; *p* ≤ 0.001), hops (−0.14 events; *p* ≤ 0.001), and aggressive encounters (−0.06 events; *p ≤* 0.05) were decreased with age (Table 4).

During the open-field test, older rabbits spent more time exploring (+23 s) and running (+2.2 s) (0.01 ≤ *p* ≤ 0.05), however they crossed the center of the arena less frequently (−0.84 times; *p* ≤ 0.05). An interaction between tube and animal age was found for time spent in biting behaviors which increased with age, especially in rabbits housed in pens with a tube compared to those in pens without (*p* ≤ 0.05) (Table 5).

## 4. Discussion

### 4.1. Effects of Enrichment Type

The elevated platform represents an additional area that animals can use and occupy to a different extent depending on different extrinsic factors (e.g., platform materials) or intrinsic ones (e.g., animal age). In the present study, rabbits were observed in the floor area in front of the platform (47.4% of total pen surface) for most of the time (50.0% of observation time on average), followed by the area under the platform (26.3% of total surface and 29.4% of observation time) and finally by the area above the platform (26.3% of total surface and 20.6% of observation time). Such a distribution of animals is consistent with previous results in collective pens (29 animals/pen) [18] that reported a higher animal density (rabbits/m^2^) on the floor compared to the platform area as well as a higher presence of them on the floor area in front of the platform than under the platform, both at every weekly observation and for the whole period (average of observations between 5 to 11 weeks of age). 

The platform design can also affect rabbit preference and use of the different pen areas. Growing rabbits kept in groups (14 animals/pen) preferred to stay under the platforms when these had a solid floor or deep-litter floor, rather than a wire-net floor [27]. Nevertheless, in the case of pens with the wire-net platforms, more rabbits stayed under platforms equipped with a fixed manure tray compared to those without. It is possible that the area under the platform with such a design may represent a hiding place, mimicking the conditions of a burrow in the wild. In addition, the material of the platform is important for growing rabbits [18] since more rabbits/m^2^ were observed on plastic-mesh platforms than on wire-mesh ones. Similarly, reproducing does and their kits at 28 days of age preferred plastic-mesh platforms over wire-mesh ones [28].

In our trial, the presence of the elevated platform had a positive effect on behavior, in terms of increased observations of comfortable resting (stretched position). The decrease of grooming in favor of biting or licking pen elements may suggest that the platform was also used to re-direct attention. These findings are in line with that of Buijs et al. [29] who showed that the presence of a wooden structure as enrichment decreased social contacts in growing rabbits kept in cages of 8 animals. Lastly, rabbit reactivity in a new environment, assessed by the open-field test, was not modified to a relevant extent by the presence of this structural enrichment. Similarly, very few behavioral differences were observed during the open-field test in adult laboratory rabbits housed in conventional cages vs. enriched cages with a box (shelter and raised height) [20].

Under the conditions of the present study, the platform did not modify rabbit growth performance, in agreement with previous research in rabbits kept at different groups sizes in collective cages (16 animals/cage, [30]) or collective pens (29 animals/pen, [17,18]; 60 animals/pen, [16]). On the other hand, and in contrast to these latter studies, we recorded a higher prevalence of injured animals at the end of the trial in pens with a platform compared to those without. In the present study, this negative result is likely due to the higher group size in the former compared to the latter pens (37 vs. 26 rabbits/pen) that was used to maintain a constant stocking density (16 rabbits/m^2^), rather than to the presence of the platform itself. In fact, in growing rabbits, aggressive behavior and related injuries have been found to increase with group size. For example, at the same stocking density of the present study, an increased occurrence of agonistic behavior from cages with 2 rabbits to pens with 13 rabbits (average across 46 and 74 days of age) was found [12]. Likewise, at 77 days of age, a significant increase in the percentage of injured rabbits (with ear lesions) was observed from cages with 2 (0.0%) or 8 rabbits (7.1%) or small pens with 10 to 13 rabbits (8.7%) to large pens with 20 to 26 rabbits (17.4%) [31]. Although the number of aggressive rabbits may be low even in large groups, these individuals can injure more pen mates when kept in large groups. To our knowledge, little is known about the dynamics of attacks at hierarchy establishment in growing rabbits kept in groups under commercial conditions. Vervaecke et al. [32] showed that growing rabbits reared in groups of 8 animals could form strong hierarchies from 10 weeks of age onwards.

In our study, the lack of agreement between aggression observed at 63 and 70 days of age and the rate of injured animals at the end of the trial could be attributed to the fact that behaviors were recorded one week before scoring for injuries. On the other hand, we could also hypothesize that the observation time (2 min every 30 min for a total of 96 min during 24 h) during the behavioral analysis may have impeded the detection of events such as aggression.

Despite the benefits mentioned above, some authors claim that elevated platforms can make some daily farm management procedures difficult (e.g., health-status check) because animals are less visible and accessible. Moreover, the hygiene in the cage/pen can also be impaired [33], especially when platforms are not located in the correct position and/or made from inadequate materials.

The inclusion of a plastic tube as a structural enrichment aimed to provide a place in which rabbits could escape from unwanted social contacts from other pen mates or could recover at the end of a flight response in case of any disturbing event, including aggression. On average, rabbits were observed inside the tube for a relatively short time period (2.4% of observation time, on average), which increased to 4.6% in those pens with only a tube and decreased to 0.25% in pens with both a platform and tube. These results are independent of the area the tube occupied within the pen (6.0% vs. 4.4% of total surface) and from the group size (26 vs. 37 rabbits/pen) in the different pen types (only tube vs. platform and tube). As indicated rabbits preferred the platform to the tube and only used the tube when no other enrichment item was available. The age at which behavioral recordings were performed (63 days or 70 days) may account for the scarce use of the tube. Young animals soon after weaning are expected to use and share the tube more frequently because they look for close contact [34], and have a smaller size than the older rabbits of the current study. Nevertheless, it could be further questioned whether the conditions for the hiding tube used in the present study (size, shape, number) were the most appropriate for the collective pens.

Literature on the use of hiding elements is scarce and mainly limited to laboratory rabbits. Hansen and Berthelsen [20] found that very few adult rabbits kept in individual cages used a box as a place for resting or escaping, whereas they more often used the top of the box as a look-out or resting place. Likewise, according to Lidfors [35], male rabbits rarely used a box to hide, and they performed the same abnormal behaviors of rabbits kept in conventional cages. Fuentes and Newgren [36] compared individual and group-housed laboratory rabbits for a period of 60 days, the latter kept in a room equipped with plastic crates and a litter box as hiding elements. Although specific behavioral observations were not made, these authors did not report aggression among animals and a low use of the box. 

In our trial, the tube did not serve as an agent of reducing the rate of injured rabbits or aggressive behavior, consistent with findings in reproducing does housed in part-time collective systems equipped with hiding places (one plastic tube and one platform) [19]. On the other hand, growth performance was negatively affected, which was not expected. Under farming conditions and collective housing, Tuyttens et al. [37] observed no substantial effect of environmental enrichment, consisting of a gnawing stick, an elevated platform and a hiding box simultaneously present in the pen (17 rabbits/pen), on growth performance over the entire period (slaughter at 63–72 days) compared to conventional pens (34 rabbits/pen). Similarly, no difference was found for daily weight gain between adult laboratory rabbits kept in enriched cages with a box and unenriched ones [35].

Under our conditions, the negative result of the tube is difficult to explain. The hypothesis of greater competition to enter and stay inside the tube at the expense of feeding and drinking, that could be further aggravated as animals grow and body size increases, is not supported by data. 

### 4.2. Effects of Animal Age

The effects of increasing slaughter age on productive performance and final weight observed in the present trial are consistent with a previous study in collective pens and large groups (20–27 rabbits/pen) and confirm previous research with individual or bicellular cages [38,39]. In the case of group housing, the growth impairment at older ages may be more pronounced than that observed under conventional housing because of the increase of aggression due to approaching sexual maturity. Szendrő et al. [31] reported a considerable increase of ear lesions associated with aggression in rabbits kept in cages (2 rabbits/cage) or pens of different sizes (6–26 rabbits/pen) from 63 to 77 days of age. Similar results were obtained by Rommers and Meijerhof [40] for several group sizes (6–54 rabbits/cage) when age increased from 73 to 80 days. In our experiment, however, the rate of injured animals did not significantly increase with age which could be explained by the relatively young age of animals compared to other studies [40], as well as by differences in housing conditions and animal genetics. Using similar collective pens and group sizes, Trocino et al. [41] did not find a significant increase in injured animals from 76 to 83 days.

Despite only one week of difference between recordings, rabbit behavioral pattern across 24 h significantly changed towards a more passive attitude (less rearing and hops, more self-grooming) and change in the resting position (more crouched body). These variations can be associated partly with the reduction of the available space per animal with increasing animal age and size. Trocino et al. [24] also observed a less active behavior and a longer time resting with crouched body in older rabbits housed in collective pens (76 days vs. 83 days).

On the other hand, during the open-field test, older rabbits increased cautious exploration and running in the new environment, but crossed the center of the arena fewer times, which can be considered to be due to a more fearful attitude. In a previous study [9], fewer displacements and more standing still events were observed at older ages, which are considered passive reactions to a new environment. These results might reflect an impairment of rabbits’ state with age when kept in large groups in a confined environment because of physiological and behavioral changes linked to their growth and sexual development. On the other hand, animal behavior during the open field test is affected by different rationales, such as fear, or motivation to explore the environment or to reinstate social contacts [42], and these factors may change with age.

## 5. Conclusions

Results of the present study illustrate that group housing of growing rabbits in pens with an elevated plastic-slatted platform allows rabbits to increase their behavioral repertoire and movement opportunities without negative effects on production traits or animal reactivity at the open field test. Nevertheless, based on the rate of injured rabbits, the higher available surface area offered by the platform and the corresponding higher group size pose great risks for social instability and related aggression among pen mates. On the other hand, the inclusion of a hiding tube impaired growth performance and did not improve rabbit welfare in terms of behavior. The underlying reasons for the negative outcomes of the hiding tube warrants further investigation to evaluate whether the structural characteristics (material, shape, size) and/or number of tubes could affect their use.

## Figures and Tables

**Table 1:** Ethogram used for behavioral evaluation of rabbits (*n* = 504) with and without environmental enrichment: location, categories and description of behaviors [22,23,24].

Location	Category	Behavior	Description
Floor	Nutrition	Feeding ^1^	Head in feeder
		Drinking ^1^	Mouth in contact with drinking nipples
Floor and/or platform	Resting	Body crouched ^1^	Inactive with body crouched and abdomen in contact with the floor
		Body stretched ^1^	Inactive with body, fore and/or hind legs stretched and in contact with the floor
		Self-grooming ^1^	Licking, nibbling, scratching the own head or body or stroking the fore legs over it
	Social	Allo-grooming ^1^	Licking or nibbling a pen mate
		Aggressive interactions ^2^	Threatening, biting, attacking, fighting, pushing, chasing or scratching other rabbit/s
	Explorative	Sniffing ^1^	Standing or moving with fore legs while sniffing around
		Biting/licking ^1^	Biting/licking elements of the pen
		Hops ^2^	Displacing the whole body with a hop
		Rearing ^2^	Standing upright on hind legs
	Locomotor	Moving ^1^	Displacing the whole body
		Running ^1^	Displacing the whole body rapidly

^1^ Measured as duration (min). ^2^ Counted as number of events.

**Table 2:** Behaviors evaluated during the open-field test [24,25,26].

Behavior	Description
Total displacements	Number of squares that the rabbit crossed in the arena
Central displacements	Number of times the rabbit crossed the square in the centre of the arena
Movement	Time spent in moving with fore and hind legs among the squares
Running	Time spent in running among the squares
Exploration	Time spent moving with forelegs or standing while sniffing and looking around inside the same square
Escape attempts	Number of rapid runs towards the corners of the arena
Hops	Number of times the rabbit completely displaced its body by a hop
Standing still	Time the rabbit spent still with its fore and hind legs unstretched and on the ground
Rearing	Number of times the rabbit reared up on its hind legs
Grooming	Time spent self-grooming
Digging	Time spent digging inside the arena
Biting	Time spent biting elements of the pen
Resting	Time spent inactive with the body touching the floor and fore and/or hind legs stretched on the floor
Defecation	Number of times the rabbit defecated
Urination	Number of times the rabbit urinated

**Table 3:** Effects of enrichment (platform or tube) and slaughter age on rabbit growth performance (least square means) from weaning (33 days) to slaughter (68 days or 75 days) for group housed rabbits.

Parameter	Platform (P) ^1^		Tube (T) ^2^		Slaughter Age (A) ^3^		*p*-Value			RSD
	No	Yes	No	Yes	68 d	75 d	P	T	A	
Pens (n)	8	8	8	8	8	8				
Rabbits (n)	214	286	251	249	250	250				
Live weight (g)										
at 33 days	975	975	974	976	974	976				4.06
at slaughter	2550	2601	2613	2539	2465	2687			***	80.2
Weight gain (g/day)	42.4	43.6	44.0	41.8	44.1	41.7		*	*	1.85
Feed intake (g/day)	147	147	149	145	144	150			***	3.89
Feed conversion	3.48	3.39	3.39	3.48	3.25	3.61			***	0.12
Injured rabbits (%)	11.7	20.6	14.7	18.9	15.6	18.0	**			-

RSD, residual standard deviation. * *p* ≤ 0.05, ** *p* ≤ 0.01, *** *p* ≤ 0.001. Values not reported where *p* > 0.05. The complete set of p-values for the single effects and their interactions is provided in Supplementary Table S1. ^1^ No: Least square means of data related to animals in pens without the platform, with and without the tube, slaughtered at 68 d and at 75 d. Yes: Least square means of data related to animals in pens with the platform, with and without the tube, slaughtered at 68 d and at 75 d. ^2^ No: Least square means of data related to animals in pens without the tube, with and without the platform, slaughtered at 68 d and at 75 d. Yes: Least square means of data related to animals in pens with the tube, with and without the platform, slaughtered at 68 d and at 75 d. ^3^ 68 d: Least square means of data related to animals slaughtered at 68 d from all types of pens. 75 d: Least square means of data related to animals slaughtered at 75 d from all types of pens.

**Table 4:** Effects of enrichment (platform or tube) and age (63 days and 70 days) on rabbit behavior (least square means ± standard error) registered across 24 h.

Parameter	Platform (P) ^1^		Tube (T) ^2^		Age (A) ^3^		*p*-Value				
	No	Yes	No	Yes	63 days	70 days	P	T	A	P × T	P × A
Rabbits (*n*)	214	286	251	249	500	250					
Nutrition behavior (% of the total observation time)											
Feeding	8.31 ± 0.29	8.22 ± 0.29	8.49 ± 0.30	8.05 ± 0.28	8.17 ± 0.20	8.37 ± 0.25					
Drinking	2.18 ± 0.12	1.72 ± 0.10	2.08 ± 0.11	1.81 ± 0.10	2.01 ± 0.08	1.87 ± 0.10	**				
Other behavior (% of time spent on these behaviors ^a^)											
Total resting	70.6 ± 0.63	73.2 ± 0.65	72.0 ± 0.64	71.8 ± 0.64	75.3 ± 0.48	68.6 ± 0.58	*		**		
Resting crouched body	34.8 ± 0.60	34.0 ± 0.58	34.3 ± 0.59	34.4 ± 0.59	31.5 ± 0.39	37.5 ± 0.55			***		
Resting stretched body ^4^	35.0 ± 0.69	38.8 ± 0.76	37.2 ± 0.73	36.5 ± 0.72	43.8 ± 0.60	31.0 ± 0.51	***		***		***
Self-grooming ^5^	19.6 ± 0.49	18.4 ± 0.47	18.6 ± 0.47	19.3 ± 0.49	16.5 ± 0.30	21.8 ± 0.45			***		***
Allo-groming	1.46 ± 0.10	0.97 ± 0.07	1.15 ± 0.08	1.23 ± 0.09	1.26 ± 0.06	1.11 ± 0.07	***		*		
Sniffing ^6^	5.52 ± 0.31	4.94 ± 0.28	5.26 ± 0.30	5.18 ± 0.29	4.39 ± 0.18	6.20 ± 0.28			***	*	
Biting/licking	0.25 ± 0.04	0.45 ± 0.06	0.38 ± 0.06	0.29 ± 0.05	0.37 ± 0.04	0.30 ± 0.04	**				
Moving ^7^	1.06 ± 0.06	0.86 ± 0.05	0.94 ± 0.06	0.98 ± 0.06	0.97 ± 0.04	0.95 ± 0.06	*			***	
Explorative and aggressive behavior (number of events per pen per 2 min)											
Rearing	0.16 ± 0.02	0.30 ± 0.03	0.28 ± 0.03	0.17 ± 0.02	0.30 ± 0.02	0.16 ± 0.02	***	**	***		
Hops	0.11 ± 0.02	0.08 ± 0.02	0.10 ± 0.02	0.09 ± 0.02	0.19 ± 0.02	0.05 ± 0.01			***		
Aggressive behavior	0.16 ± 0.03	0.05 ± 0.02	0.07 ± 0.02	0.10 ± 0.02	0.12 ± 0.02	0.06 ± 0.02	**		*		

* *p* ≤ 0.05, ** *p* ≤ 0.01, *** *p* ≤ 0.001. Values not reported when *p* > 0.05. The complete set of p-values for the single effects and their interactions is provided in Supplementary Table S2. ^a^ Excluding the time spent feeding, drinking, under the platform and/or inside the tube. ^1^ No: Least square means of data related to animals in pens without the platform, with and without the tube, recorded at 63 d and at 70 d. Yes: Least square means of data related to animals in pens with the platform, with and without the tube, recorded at 63 d and at 70 d. ^2^ No: Least square means of data related to animals in pens without the tube, with and without the platform, recorded at 63 d and at 70 d. Yes: Least square means of data related to animals in pens with the tube, with and without the platform, recorded at 63 d and at 70 d. ^3^ 63 d: Least square means of data recorded at 63 d from all types of pens. 75 d: Least square means of data recorded at 70 d from all types of pens. ^4^ Interaction P × A, *p* ≤ 0.001; resting with stretched body: 42.7% in pens without platform at 63 days, 45.0% in pens with platform at 63 days, 28.7% in pens without platform at 70 days and 33.5% in pens with platform at 70 days. ^5^ Interaction P × A, *p* ≤ 0.001; self-grooming: 17.6% in pens without platform at 63 days, 15.5% in pens with platform at 63 days, 21.8% in pens without platform at 70 days and 21.9% in pens with platform at 70 days. ^6^ Interaction P × T, *p* ≤ 0.05; sniffing: 6.12% in pens with no enrichment and 4.52% in pens with platform, 4.97% in pens with tube, and 5.40% in pens with platform and tube. ^7^ Interaction P × T, *p* ≤ 0.001; moving: 1.20% in pens with no enrichment, and 0.73% in pens with platform, 0.94% in pens with tube, and 1.02% in pens with platform and tube.

**Table 5:** Effects of enrichment (platform or tube) and age (65 days and 72 days) on the reactivity (least square means ± standard errors) of rabbits during the open-field test.

Parameter	Platform (P) ^1^		Tube (T) ^2^		Age (A) ^3^		*p*-Value				
	No	Yes	No	Yes	65 days	72 days	P	T	A	P × T	T × A
Rabbits (*n*)	80	80	80	80	80	80					
Entered animals (%) ^a^	87.5	78.8	78.8	87.5	83.8	82.5					
Latency (s)	19.8 ± 2.45	23.9 ± 2.95	21.0 ± 2.78	22.5 ± 2.60	21.1 ± 2.60	22.4 ± 2.77					
Displacements (*n*)											
Total	49.7 ± 2.24	52.3 ± 2.35	52.4 ± 2.35	49.6 ± 2.24	48.2 ± 2.18	53.9 ± 2.41					
Central	3.88 ± 0.24	3.75 ± 0.24	3.95 ± 0.23	3.68 ± 0.24	4.25 ± 0.25	3.41 ± 0.22			*		
Exploration (s)	516 ± 5.04	529 ± 5.16	519 ± 5.07	526 ± 5.13	511 ± 5.00	534 ± 5.20			**		
Movement (s)	39.2 ± 1.58	39.2 ± 1.58	39.9 ± 1.61	38.5 ± 1.56	38.0 ± 1.54	40.4 ± 1.63					
Running (s)	5.79 ± 0.61	7.24 ± 0.75	7.30 ± 0.76	5.74 ± 0.61	5.48 ± 0.58	7.66 ± 0.79			*		
Standing still (s)	51.8 ± 3.82	50.8 ± 3.75	53.7 ± 3.99	49.0 ± 3.62	46.9 ± 3.47	56.1 ± 4.13					
Grooming (s) ^4^	5.87 ± 0.66	8.05 ± 0.88	6.40 ± 0.71	7.37 ± 0.81	6.75 ± 0.75	6.99 ± 0.77	*			***	
Biting (s) ^5^	5.42 ± 1.49	6.21 ± 1.70	6.71 ± 1.83	5.01 ± 1.39	2.87 ± 0.80	11.7 ± 3.18			***		*

* *p* ≤ 0.05, ** *p* ≤ 0.01, *** *p* ≤ 0.001. Values not reported when *p* > 0.05. The complete set of p-values for the single effects and their interactions is provided in Supplementary Table S3. ^a^ Rabbits that entered the pen spontaneously within a period of 60 s. ^1^ No: Least square means of data related to animals in pens without the platform, with and without the tube, recorded at 65 d and at 72 d. Yes: Least square means of data related to animals in pens with the platform, with and without the tube, recorded at 65 d and at 72 d. ^2^ No: Least square means of data related to animals in pens without the tube, with and without the platform, recorded at 65 d and at 72 d. Yes: Least square means of data related to animals in pens with the tube, with and without the platform, recorded at 65 d and at 72 d. ^3^ 65 d: Least square means of data recorded at 65 d from all types of pens. 72 d: Least square means of data recorded at 72 d from all types of pens. ^4^ Interaction P×T, *p* ≤ 0.001; grooming, 4.16 s in in pens with no enrichment, 9.85 s in pens with platform, 8.25 s in pens with tube, and 6.58 s in pens with platform and tube. ^5^ Interaction T × A, *p* ≤ 0.05; biting, 5.26 s in pens without tube at 65 d, 1.56 s in pens with tube at 65 d, 8.55 s in pens without tube at 72 d, and 16.1 in pens with tube at 72 d.

## Supplementary Materials

The following are available online at https://www.mdpi.com/2076-2615/9/8/537/s1: Table S1: P-values of the effects of platform (P), tube (T), slaughter age (A), and their interactions on rabbit growth performance from weaning (33 days) to slaughter (68 days or 75 days), Table S2: P-values of the effects of platform (P), tube (T), animal age (A), and their interactions on rabbit behavior registered across 24 h, Table S3: P-values of the effects of platform (P), tube (T), animal age (A), and their interactions on the reactivity of rabbits during the open-field test, Table S4: Descriptive statistics of rabbit behavior registered across 24 h according to the presence (no or yes) of enrichments (platform and tube) and animal age (63 or 70 days): raw mean (M), standard deviation (SD), minimum (Min) and maximum (Max), Table S5: Descriptive statistics of reactivity of rabbits during the open-field test according to the presence (no or yes) of enrichments (platform and tube) and animal age (65 or 72 days): raw mean (M), standard deviation (SD), minimum (Min) and maximum (Max).
