# Supplementary material for: The Use of Environmental Enrichments Affects Performance and Behavior of Growing Rabbits Housed in Collective Pens
Source: Animals (Basel). 2019 Aug 7;9(8):537. doi: 10.3390/ani9080537 (PMC6720832; doi:10.3390/ani9080537)
Supplement: Supplementary file 1 [file animals-09-00537-s001.pdf]

Table S1. P-values of the effects of platform (P), tube (T), slaughter age (A), and their interactions on rabbit growth performance from weaning (33 days) to slaughter (68 days or 75 days).

Parameter	P	T	A	P×T	P×A	T×A	P×T×A
Live weight at 33 days (g)	0.857	0.354	0.528	0.818	0.148	0.753	0.468
Live weight at slaughter	0.239	0.105	0.001	0.727	0.879	0.247	0.508
Weight gain (g/day)	0.294	0.045	0.032	0.852	0.656	0.390	0.584
Feed intake (g/day)	0.989	0.072	0.008	0.159	0.682	0.343	0.693
Feed conversion	0.171	0.161	< 0.001	0.315	0.680	0.065	0.622
Injured rabbits (%)	0.003	0.340	0.083	0.100	0.094	0.257	0.317

Table S2. P-values of the effects of platform (P), tube (T), animal age (A), and their interactions on rabbit behavior registered across 24 h.

Parameter	P	T	A	P×T	P×A	T×A	P×T×A
Feeding [1]	0.826	0.284	0.304	0.419	0.060	0.119	0.876
Drinking [1]	0.002	0.076	0.141	0.889	0.725	0.668	0.824
Total resting [2]	0.040	0.766	0.005	0.146	0.779	0.565	0.621
Resting crouched body [2]	0.314	0.937	< 0.001	0.956	0.432	0.853	0.262
Resting stretched body [2]	< 0.001	0.488	< 0.001	0.388	< 0.001	0.652	0.439
Self-grooming [2]	0.084	0.268	< 0.001	0.937	< 0.001	0.062	0.122
Allo-groming [2]	< 0.001	0.501	0.047	0.158	0.196	0.543	0.436
Sniffing [2]	0.166	0.839	< 0.001	0.017	0.459	0.124	0.753
Biting/licking [2]	0.006	0.210	0.077	0.797	0.475	0.088	0.249
Moving [2]	0.016	0.613	0.762	< 0.001	0.866	0.459	0.110
Rearing [3]	< 0.001	0.004	< 0.001	0.706	0.784	0.092	0.453
Hops [3]	0.288	0.679	< 0.001	0.177	0.689	0.237	0.190
Aggressive behavior [3]	0.001	0.338	0.027	0.066	0.438	0.747	0.435

[1] % of the total observation time; [2] % of time spent on these behaviors by excluding the time spent feeding, drinking, under the platform and/or inside the tube; [3] number of events per pen per 2 min.

Table S3. P-values of the effects of platform (P), tube (T), animal age (A), and their interactions on the reactivity of rabbits during the open-field test.

Parameter	P	T	A	P×T	P×A	T×A	P×T×A
Entered animals (%)	0.154	0.108	0.933	0.617	0.617	0.777	0.155
Latency (s)	0.282	0.689	0.717	0.668	0.582	0.668	0.317
Total displacements (n)	0.418	0.400	0.085	0.288	0.908	0.909	0.801
Central displacements (n)	0.703	0.418	0.013	0.166	0.609	0.347	0.076
Exploration (s)	0.057	0.323	0.001	0.186	0.721	0.647	0.228
Movement (s)	0.992	0.556	0.276	0.064	0.723	0.263	0.241
Running (s)	0.134	0.107	0.025	0.944	0.885	0.261	0.361
Standing still (s)	0.857	0.391	0.091	0.416	0.559	0.466	0.352
Grooming (s)	0.045	0.368	0.824	< 0.001	0.068	0.836	0.975
Biting (s)	0.725	0.455	< 0.001	0.198	0.779	0.018	0.680

Table S4. Descriptive statistics of rabbit behavior registered across 24 h according to the presence (no or yes) of enrichments (platform and tube) and animal age (63 or 70 days): raw mean (M), standard deviation (SD), minimum (Min) and maximum (Max).

	Platform								Tube								Age							
	No				Yes				No				Yes				63 days				70 days			
	M	SD	Min	Max	M	SD	Min	Max	M	SD	Min	Max	M	SD	Min	Max	M	SD	Min	Max	M	SD	Min	Max
Feeding [1]	8.41	6.61	0.00	30.8	8.13	5.47	0.00	26.9	8.43	6.20	0.00	30.8	8.11	5.93	0.00	27.9	8.17	6.09	0.00	30.8	8.47	6.02	0.00	29.6
Drinking [1]	2.16	2.53	0.00	12.7	1.74	2.12	0.00	13.9	2.09	2.43	0.00	13.9	1.81	2.24	0.00	12.7	2.03	2.42	0.00	13.9	1.79	2.18	0.00	11.1
Total resting [2]	71.8	13.8	25.0	100	74.4	13.5	35.2	99.2	73.2	13.9	31.78	99.2	72.9	13.4	25.0	100	75.4	13.6	25.0	100	68.5	12.7	31.96	97.4
Resting crouched body [2]	33.6	13.0	0.51	72.9	33.4	12.7	1.04	72.9	33.2	12.1	0.96	72.5	33.8	13.5	0.51	72.9	31.5	12.4	0.51	73.0	37.5	12.8	5.59	72.9
Resting stretched body [2]	38.2	17.9	0.00	92.5	41.0	16.6	0.00	90.2	40.1	16.7	0.00	92.5	39.1	17.9	0.00	91.4	43.9	17.1	0.00	92.5	31.1	14.5	0.00	79.8
Self-grooming [2]	19.1	10.2	0.00	59.3	17.7	9.92	0.00	56.3	18.3	10.1	0.00	53.0	18.5	10.1	0.00	59.3	16.5	9.61	0.00	56.3	22.1	9.98	1.01	59.3
Allo-groming [2]	1.48	1.95	0.00	12.5	1.02	1.88	0.00	22.8	1.27	2.02	0.00	22.8	1.23	1.83	0.00	12.5	1.29	1.94	0.00	22.8	1.18	1.91	0.00	12.5
Sniffing [2]	5.08	6.23	0.00	41.8	4.77	5.38	0.00	26.7	4.86	6.15	0.00	41.7	4.99	5.47	0.00	41.5	4.41	5.42	0.00	41.8	5.96	6.43	0.00	41.5
Biting/licking [2]	0.26	0.97	0.00	11.7	0.48	1.43	0.00	14.6	0.40	1.37	0.00	14.6	0.33	1.07	0.00	8.08	0.39	1.25	0.00	11.7	0.32	1.19	0.00	14.6
Moving [2]	1.08	1.37	0.00	11.7	0.88	1.17	0.00	16.2	0.95	1.21	0.00	10.5	1.01	1.34	0.00	16.2	0.97	1.22	0.00	11.7	0.99	1.39	0.00	16.2
Rearing [3]	0.19	0.50	0	3	0.35	0.79	0	10	0.32	0.76	0	10	0.22	0.55	0	4	0.31	0.65	0	4	0.19	0.69	0	10
Hops [3]	0.18	0.55	0	5	0.12	0.41	0	4	0.14	0.45	0	4	0.16	0.52	0	5	0.20	0.55	0	5	0.05	0.30	0	3
Aggressive behavior [3]	0.18	0.70	0	8	0.07	0.32	0	4	0.11	0.45	0	5	0.15	0.63	0	8	0.14	0.62	0	8	0.09	0.35	0	3

[1] % of the total observation time; [2] % of time spent on these behaviors by excluding the time spent feeding, drinking, under the platform and/or inside the tube; [3] number of events per pen per 2 min.

Table S5. Descriptive statistics of reactivity of rabbits during the open-field test according to the presence (no or yes) of enrichments (platform and tube) and animal age (65 or 72 days): raw mean (M), standard deviation (SD), minimum (Min) and maximum (Max).

	Platform								Tube								Age							
	No				Yes				No				Yes				65 days				75 days			
	M	SD	Min	Max	M	SD	Min	Max	M	SD	Min	Max	M	SD	Min	Max	M	SD	Min	Max	M	SD	Min	Max
Entered (%) [1]	87.5	-	-	-	78.8	-	-	-	78.8	-	-	-	87.5	-	-	-	83.8	-	-	-	82.5	-	-	-
Latency (s)	20.0	12.6	2	57	23.7	15.5	3	59	21.1	13.3	2	59	22.2	14.8	3	59	21.2	13.8	4	59	0.18	0.38	0	1
Total (n) [2]	49.7	21.4	2	109	52.5	23.0	1	134	52.6	21.9	2	99	49.7	22.5	1	134	48.3	21.8	2	109	22.2	14.4	2	57
Central (n) [3]	3.91	2.86	0	12	3.84	2.97	0	12	3.99	2.98	0	12	3.76	2.86	0	12	4.28	3.03	0	12	54.0	22.3	1	134
Exploration (s)	516	43.8	430	623	530	69.7	412	966	519	45.9	412	696	527	68.9	430	966	511	45.7	412	725	3.48	2.75	0	10
Movement (s)	39.5	15.1	1	75	39.6	15.9	0	84	40.3	16.2	1	84	38.8	14.7	0	75	38.3	16.3	1	84	535	67.1	455	966
Running (s)	5.91	8.32	0	42	7.49	10.6	0	56	7.40	9.41	0	42	6.00	9.62	0	56	5.65	8.57	0	42	40.8	14.6	0	79
Standing still (s)	53.0	33.8	6	157	51.3	25.8	5	128	54.7	30.4	6	157	49.5	29.6	5	154	47.3	27.6	6	128	7.75	10.3	0	56
Grooming (s)	6.33	8.36	0	43	8.40	11.6	0	68	7.16	10.9	0	68	7.56	9.35	0	43	7.09	8.74	0	43	57.0	31.7	5	157
Biting (s)	8.49	20.0	0	100	8.14	16.9	0	75	7.33	15.9	0	76	9.30	20.8	0	100	3.55	13.4	0	76	7.64	11.4	0	68

[1] Rabbits that entered the pen spontaneously within a period of 60 s; [2] Total displacements; [3] Central displacements.
